# Rush Medical College

**Published:** 1869-07-15

**Authors:** 


### EDITORIAL.

### *Rush Medical College.*

The Annual Catalogue and announcement of the course for 1869 and 1870 will be issued soon. Correspondents please notice.

